# Nurses and Physicians’ Perceptions Regarding the Role of Oncology Clinical Nurse Specialists in an Exploratory Qualitative Study

**DOI:** 10.3390/healthcare11131831

**Published:** 2023-06-22

**Authors:** Keren Dopelt, Noam Asna, Mazal Amoyal, Osnat Bashkin

**Affiliations:** 1 Department of Public Health, Ashkelon Academic College, Ashkelon 78211, Israel; osnatba@edu.aac.ac.il; 2 School of Public Health, Faculty of Health Sciences, Ben Gurion University of the Negev, Beer Sheva 84105, Israel; 3 Oncology Institute, Shaare Zedek Medical Center, Jerusalem 91031, Israel; asnaoffice@yahoo.com; 4 Palliative Care Unit, Barzilai Medical Center, Ashkelon 78306, Israel; mazalam@bmc.gov.il

**Keywords:** oncology, nursing, clinical nurse specialists, cancer patients, expanding nurses’ authority, professionalization, policy, education

## Abstract

The purpose of the study was to examine the attitudes of nursing and medical teams about the role of oncology clinical nurse specialists in the healthcare system in Israel, where, unlike many countries in the world, such a role has not yet been developed or professionally defined. We conducted 24 interviews with physicians and nurses between August and October 2021. The interviews were transcribed and analyzed using a thematic analysis method. The Consolidated Criteria for Reporting Qualitative Research checklist was used to report the study. Five main themes emerged from the interviews: (1) contribution to the healthcare system, (2) contribution to the patient, (3) drawing professional boundaries, (4) additional responsibilities and authority for oncology clinical nurse specialists, and (5) the field’s readiness for a new position of oncology clinical nurse specialists. The findings provide evidence about the need to develop the role of clinical nurse specialists in the oncology field due to its potential benefits for nurses, physicians, patients, family members, and the healthcare system. At the same time, an in-depth exploration of the boundaries of the role and its implementation, in full cooperation with the oncologists and relevant professional unions, is needed to prevent unnecessary conflicts in the oncology field. Professional development training programs in nursing must create a platform for open dialogue between key stakeholders, nurses, and physicians, in order to help all involved parties, place the benefits to the patients above any personal or status considerations.

## 1. Introduction 

Israel has approximately 200,000 cancer patients, with some 29,000 new cases annually [1]. The oncology field involves complex clinical treatments and deals with complicated psychosocial issues associated with patients and their family members [2]. In recent decades, countries throughout the world have developed medical support positions, such as clinical nurse specialists, as a strategy to meet the healthcare system’s challenges. Studies show that clinical nurse specialists can provide the necessary medical care in situations meeting the position’s definitional framework and, in a manner, offering the optimal response to patients’ needs [3,4,5]. In the United States, there are about 300,000 clinical nurse specialists, and in the United Kingdom, about 3300, whereas in Israel, there are only 358 clinical nurse specialists working in the fields of supportive care (102), geriatrics (80), diabetes (32), surgery (30), premature infants (28), pain (8), rehabilitation (4), and policy and administration (74) [6]. 

There are barriers and disagreements regarding the definition, authority, and recognition of this role in Israel’s healthcare system, which make it difficult to expand it to other clinical areas [7]. Oncology nursing is a challenging and evolving profession that requires regular updating about both the medical aspects of the disease and the mental and social factors related to its diagnosis and treatment [8]. The oncology clinical nurse specialist can improve patients’ health outcomes and quality of life indicators [9] and thereby increase patients’ satisfaction with the treatment and involvement in disease management [10,11]. Moreover, integrating clinical nurse specialists leads to decreased rates of hospitalization, mortality, and complications [12]. 

A recent study in Israel examined the experiences of 39 clinical nurse specialists in supportive care. The nurses reported dissatisfaction with the work environment and with how their role was recognized and implemented by hospital physicians and managers. In addition, the limited authority they were granted did not correspond to the description of the role [13]. These findings are consistent with the results of a previous study conducted in Canada, which found barriers to the implementation and assimilation of clinical nurse specialists, including the lack of a model to guide the implementation of the role, lack of an agreed-upon description of the role and responsibilities; and lack of ongoing support and mentorship [14].

Despite the positive evidence of the inherent benefits of oncology clinical nurse specialists, there is disagreement about the role’s definition and necessity. A study that examined the perceptions of this role in the field of oncology found that definitions of the position were unclear. While physicians and managers perceived the role of an oncology clinical nurse specialist as “helping” medical practitioners in managing their workloads, the oncology clinical nurse specialists themselves perceived their role as promoting holistic, patient-centered care and proactively meeting the unique oncology patients’ needs [11]. Conflicts concerning the boundaries of the role, lack of resources and organizational and systemic support, and physicians’ fear that clinical nurse specialists will replace them limit the potential of the role and reduce its essential contribution to quality care in oncology [11].

The growing number of cancer patients and their multiple needs, the shortage of oncologists, and the rapid changes in the clinical, organizational, and technological environment in the field of oncology highlight the need to update the clinical and managerial skills of oncology nurses. The literature shows that oncology clinical nurse specialists offer many advantages. However, in contrast to many countries around the world (the United States, Canada, the United Kingdom, Japan, Brazil, and others), in Israel, the position of an oncology clinical nurse specialist has not yet been established. Implementation of this role in the healthcare system in Israel depends to a large extent on a characterization of the position, understanding its benefits for the healthcare system and the patients, and an in-depth understanding of the barriers to its implementation that must be taken into account already at the planning stage. The purpose of the present study is to examine the potential contribution of oncology clinical nurse specialists as seen through the eyes of medical and nursing professionals.

## 2. Methods

We conducted an exploratory qualitative study using semi-structured interviews. The study was approved by the Ashkelon Academic College Ethics Committee (Approval No. 20-2020). The Consolidated Criteria for Reporting Qualitative Research (COREQ) checklist was used to report the study.

### 2.1. Population and Procedure

Semi-structured in-depth interviews were conducted between August and October 2021, after informed consent was obtained from twenty-four healthcare professionals from various medical centers in all geographical parts of Israel, using purposeful sampling. Purposeful sampling is a non-random sampling technique that uses specific criteria or purposes to select a sample [15]. The aim is to collect in-depth information from the right respondents. The inclusion criteria: oncology doctors or nurses, doctors or nurses in the position of decision-makers, or clinical nurse specialists. We continued the interviews until theoretical saturation was reached. To recruit interviewees, we contacted people working in the field of oncology and invited them to take part in the study. We asked each participant to provide the name of a colleague who would be willing to participate in the research. Among the twenty-four interviewees, six were physicians and eighteen nurses. Seven interviewees were males, and seventeen were females. All interviews were conducted over the telephone due to COVID-19 social distancing restrictions and were audiotaped and transcribed verbatim in Hebrew. It was emphasized to the interviewees that their details would remain confidential, no findings would be published under their name, and they did not have to answer all the questions, or they could stop the interview. The interviewer was a Clinical Psychology graduate student, trained in qualitative research methods and supervised by KD and OB. There was no relationship between the interviewer and the participants. The interviews lasted between 30 and 50 min. 

### 2.2. Study Tool

The in-depth interview guide was developed in collaboration with oncology staff members and drew on our literature reviews. The interview guide was validated using the content validation method by two oncology nurses and two physicians to ensure that the questions were relevant to the study goals. The guide was pilot tested with one oncology nurse to ensure a smooth interview flow and verify comprehension of the questions. Information collected during the interviews included perceptions toward the role of nurses in cancer care and the need to develop oncology clinical nurse specialists training (Appendix A).

### 2.3. Data Analysis

The interviews were transcribed accurately by a professional and analyzed using a thematic analysis method based on grounded theory [16] in the ATLAS.ti v.8 software (Berlin, Germany). The Grounded Theory method uses both an inductive and a deductive approach to theory development. Our analysis included incorporating deductive themes arising from the research topics and based on a literature review of quality in cancer care and cancer survivors’ needs, together with inductive themes that emerged from the data [17]. The interpretive analysis was done close to the interviews in several stages: (1) the interviews were read at least once by KD and OB to gain in-depth knowledge of the data. (2) KD and OB identified ideas, categories, and themes related to the study’s objectives. (3) central themes were redefined to include encoded quotes and examples based on re-reading the transcripts. Relevant passages were marked and allocated to one of the content themes. The themes and quotes were examined iteratively by all authors and documented in English at the final stage. The description of the findings was accompanied by citations from the interviewees and thus provided continuous evidence for matching the interpretation and the interviewees’ unique voices.

## 3. Results

### 3.1. Participants’ Characteristics

Participants’ characteristics and codification are available in Table 1.

### 3.2. Main Themes

**Figure 1:**
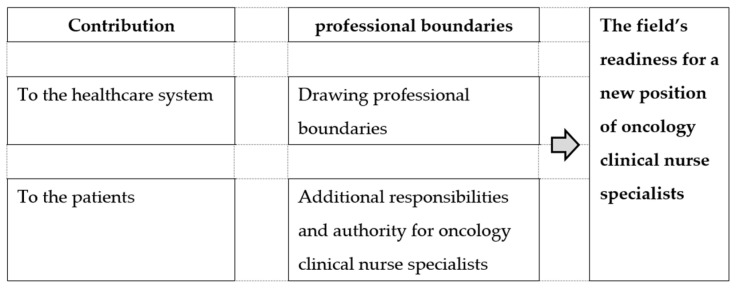
A conceptual map of the main categories.

Five main themes emerged from the interviews: (1) contribution to the healthcare system, (2) contribution to the patient, (3) drawing professional boundaries, (4) additional responsibilities and authority for oncology clinical nurse specialists, (5) the field’s readiness for a new position of oncology clinical nurse specialists. A conceptual map of the main categories is presented in Figure 1.

The themes, with quotes that illustrate each, are presented in Table 2, Table 3, Table 4, Table 5 and Table 6. 

**Theme 1—Contribution to the healthcare system.**

Nurses and physicians described multiple benefits of oncology clinical nurse specialists to the healthcare system:

1. Reducing and alleviating the burden on physicians;
2. Improving service and treatment for patients;
3. Reducing hospitalizations;
4. Creating a professional role at an intermediate level that can help patients;
5. Providing a perspective that advances the profession of nursing.

However, some physicians argue that the solution to the workforce problem is to add physicians and not to transfer responsibilities to nurses.

**Theme 2—Contribution to the patient.**

In terms of contribution to the patient, interviewees mentioned a response that is holistic and available and reduces bureaucracy and waiting time. It is particularly important to integrate the role of clinical nurse specialists into community healthcare settings and to improve services and support for convalescents. The nurse will maintain continuity in the transition between the hospital and the community. This can reduce the need for hospitalization to receive further treatment and alleviate the burden on physicians so that most treatment for convalescents will occur in community settings.

**Theme 3—Drawing professional boundaries.**

Clinical nurse specialists in various fields expressed concern about the Ministry of Health’s unwillingness to grant real responsibility to clinical nurse specialists. They also described dilemmas regarding nurses’ willingness to take on the responsibility of managing treatments or administering medications.

According to the physicians’ perception:

1. In practice, nurses know how to recommend the proper pain relief medications, but do not have the authority to prescribe them. A physician must make referrals for tests and approve the administration of any medications because, ultimately, the physician is responsible for the patient;
2. Creating a role for clinical nurse specialists unnecessarily “wastes” the nursing workforce. A nurse does not need to be a physician’s assistant. For this purpose, paramedics, for example, can be trained;
3. Politically, the process of creating a new role with expanded responsibilities must be coordinated with the physicians’ unions so that it will have their support and recognition and not be perceived as “eroding” the physicians’ role. There is a fear that physicians’ status will change as a result, or that there will be discomfort regarding the quality of nursing personnel in the healthcare system;
4. In terms of administrative hierarchy, there is a question as to whether clinical nurse specialists will be subordinate to the director of the nursing department or to the head physician of the relevant department since the clinical nurse specialist has authority similar to that of a physician.

**Theme 4—Additional responsibilities and authority for oncology clinical nurse specialists.**

Nurses suggested expanding the responsibilities of oncology clinical nurse specialists to include:

1. Prescribing pain-relieving medications;
2. Giving referrals for tests (i.e., blood tests, imaging);
3. Interpreting test results for patients;
4. Giving referrals to other professionals (nutritionists, pharmacists, etc.);
5. Participating in making therapeutic decisions;
6. Managing the treatment according to structured protocols;
7. Assisting the patient during the transition back into the community;
8. Calling the patient/family with updates.

Physicians suggested that the position of OCNS should include the following:

1. Providing a holistic response to patients, especially in terms of follow-up and transition back into the community
2. Providing long-term follow-up for patients after the intensive treatment; this would be a unique added value of the position of administering pain-relieving medications.

**Theme 5—Conflicts of oncology clinical nurse specialists’ professional status.**

Oncology nurses raised concerns about how physicians would accept oncology clinical nurse specialists. Clinical nurse specialists in other fields mentioned the gaps between the job definition compared to their actual responsibilities and the current situation in the field. The main gap pertained to prescriptions for medications given by nurse specialists, which the Pharmacists Ordinance does not recognize. So, despite their professional knowledge and experience in giving prescriptions, their authority is not recognized in practice. Another issue is that the nurses’ responsibilities are not being implemented, making it difficult to grant them more extensive responsibilities. Clinical nurse specialists described the challenges in the implementation process and gaining recognition of their role by the physicians. All nurses mentioned the importance of recognition of this role by physicians.

Physicians referred to the importance of implementing and defining the role in order to promote cooperation in the workplace so that more physicians will recognize clinical specialists as having the knowledge and authority to give advice and as people who can offer teaching and training.

## 4. Discussion

The current study aimed to examine the perceptions of nursing and medical teams about the role of oncology clinical nurse specialists. The findings reveal a complex picture regarding the OCNS role and the need for expanding nurses’ authority. The delegation of authority from physicians to nurses represents one of the most important elements in the professionalization process of nursing [18] and expanding nurses’ authority is a significant contributor to professional autonomy [19]. Various studies describe positive attitudes of physicians and nurses toward expanding nurses’ authority in several areas based on their belief that this will improve the quality of care [20,21,22]. 

The oncology nurses, some of the clinical nurse specialists, the nurses from the Ministry of Health nursing management, and the oncology physicians were unanimous about the need for oncology clinical nurse specialists’ role and about the ability of the nurses to serve as case managers. Nurses related that they see the development of an oncology clinical nurse specialist as an opportunity for professional development, especially in community healthcare settings. From an oncologist’s perspective, oncology clinical nurse specialists provide a reliable, professional workforce that can relieve their burden and improve the quality of service to the patient. 

Our findings are consistent with other studies conducted around the world indicating the importance of the oncology clinical nurse specialists in several aspects: improving cancer diagnosis and treatment services [23]; preventing the need for hospitalization and emergency services [23,24]; reducing hospitalizations [25]; issuing faster and more accurate therapeutic prescriptions; providing a reliable, accessible, and available source of information [26]; and providing psychosocial support for patients and their family members [27,28].

According to interviewees, the oncology clinical nurse specialist role can offer the added value of comprehensive treatment because currently, no single healthcare professional performs the function of providing overall management of the treatment. Similarly, Griffiths [29] reported that oncology clinical nurse specialists view the treatment of cancer patients from a holistic perspective. Brooten [30] finds an economic rationale for expanding the authority of oncology clinical nurse specialists because they provide high-quality care while potentially reducing healthcare’s high costs, given that they earn significantly lower wages than physicians. 

In the United Kingdom, clinical nurse specialists provide care once performed by physicians (prescribing medications, making diagnoses), thereby reducing the burden on physicians [31], shortening waiting times for receiving oncology services, and making treatment accessible to patients who live in peripheral areas [32]. Since 2010, the Australian government has been operating rural oncology clinics managed by oncology clinical nurse specialists to bridge gaps in access to oncology services between big cities and remote areas [9,33]. Therefore, a training and implementation model for clinical nurse specialists in oncology will empower nurses, benefit patients, reduce healthcare costs, and relieve the burden on oncology physicians, especially in peripheral areas suffering from a lack of physicians.

Despite all the inherent advantages and potential of the oncology clinical nurse specialists, some of the clinical specialist nurses and physicians from professional organizations thought that such a position is not necessary, and that even if extra assistance is needed, it can be provided by physician assistants (for example, paramedics who will undergo appropriate training) and not necessarily by a clinical nurse specialist nurse. Reasons given for this approach included: possible erosion of physicians’ status; physicians not recognizing the role and broad responsibilities of clinical nurse specialists; objections to compromising and accepting fewer professional personnel rather than increasing the number of physicians; and ambiguity regarding the role and the need for a clear and precise definition its responsibilities. Oncology nurses also raised concerns about a lack of recognition of the role on the part of the doctors. The scientific literature frequently mentions topics such as tension with other professionals, intruding on the responsibilities of other professionals in a way that harms teamwork, and ambiguity of the role of clinical specialists working in a multidisciplinary team [34]. Other studies have found that the main challenges in implementing this role are a poor understanding of it among decision-makers, lack of clarity about the role, lack of support from management, and misunderstanding of it among the medical staff [35,36,37]. Additionally, previous studies have documented condemnations of the role and criticisms of inappropriate and wasteful use of nursing personnel [27]. All these reasons mentioned in the interviews in the current study and in previous studies indicate that interviewees and researchers in the field agree that it is necessary to define clear responsibilities for the clinical nurse specialists and the maximum limits of the role’s authority [27,38].

Given the global shortage of medical and nursing staff, the World Health Organization (WHO) stated in the Munich Declaration [39] that healthcare systems must develop new roles for nurses working in hospitals and in the community. The interviewees in the current study said they think that the new role is crucial for community healthcare. Many cancer patients are treated in community healthcare settings, and cancer survivors need treatment and follow-up care in the community. Continuity between treatment in hospitals and community healthcare clinics has a considerable effect on oncology patients. Studies show that such continuity is linked to high patient satisfaction, improved quality of life and mental health indicators [40,41], improved responsiveness to treatment, and better therapist-patient communication [42]. In contrast, lack of treatment continuity was found to be related to increases in the use of unnecessary medical services [43], hospitalizations, and visits to emergency medical facilities [42].

Cancer requires complex treatment, the use of different sections of the healthcare system, and multiple caregivers. Patients and their families frequently report a lack of information concerning treatments, professionals, ways to communicate with healthcare providers, and above all, how to navigate the healthcare system [44]. Oncology clinical nurse specialists can fill this vacuum and play a key role in facilitating cancer patients’ encounters with the system. Support for this role was found both in research in the field of oncology and in studies that examined managing chronic care by nurses [45,46,47,48].

### Study Limitations

The sample is limited but is considered reasonable for exploratory studies using qualitative research methodology [49]. We made efforts to include a wide range of stakeholders related to the research topic from various settings and regions in Israel in order to obtain responses from a broad and diverse swath of the healthcare system in Israel. Moreover, the interviews were transcribed from Hebrew, the native language of Israel. This may have increased the chances for variations in the interpretation of our data. We made all efforts to ensure methodological rigor and validity of the translations from Hebrew to English by using a standardized codebook, meeting frequently, sharing and comparing our results, and performing a pilot analysis. Throughout the study, we conducted an internal quality audit during our meetings, adapted from Tong et al. [50], to determine whether the data were collected, analyzed, and reported consistently according to the study protocol.

## 5. Conclusions

Multidisciplinary, coordinated, and holistic treatment may respond to the psychosocial and clinical issues facing the oncology field. The findings of this study provide evidence about the need to develop a new role of oncology clinical nurse specialists in Israel due to its potential benefits for nurses, physicians, patients, family members, and the healthcare system as a whole. At the same time, the conclusions drawn from the study reveal a complex challenge. An in-depth exploration of the boundaries of the role and its implementation, in full cooperation with the oncologists and relevant professional unions, is needed to prevent unnecessary conflicts in the oncology field. Professional development training programs in the nursing field must create a platform for dialogue between management and key stakeholders of nursing and medical departments in order to help all involved parties place the benefits to the patients first, and above any personal or status considerations. The role of oncology clinical nurse specialists can potentially impact the quality of care, prevent hospitalizations, alleviate the pressure and burden on physicians, and reduce costs for the healthcare system. In addition, we recommend extending the responsibilities of oncology nurses to those that exist in various countries around the world (e.g., Germany, Australia, the United States, etc.) and formally designating them as treatment managers. Additionally, we recommend that more nurses be available in the community to provide support, companionship, and follow-up to cancer survivors. Based on these findings, we recommend further research examining cancer patients’ attitudes toward this suggested new role in oncology nursing in Israel.

## Figures and Tables

**Table 1:** Characteristics of the interviewees (P = Physician, N = Nurse).

Code	Gender	Role
P1	Male	Hospital Oncologist, Head of the Oncology department
P2	Male	Community family physician, Member of the National Secretariat of the Israel Medical Association (IMA)
P3	Female	Hospital Oncologist
P4	Male	Hospital and Community Oncologist, Head of the Oncology department
P5	Male	Hospital and Community Oncologists
P6	Male	Public Health physician, Board member of the Organization of State Employee Physicians, IMA
N1	Female	Senior nurse, working at the Department of professional development in Nursing Management, the Ministry of Health
N2	Female	Hospital Oncology nurse
N3	Female	Community Oncology nurse
N4	Female	Community Palliative nurse
N5	Female	Community Palliative nurse
N6	Female	Community Palliative and Oncology nurse
N7	Female	Hospital Oncology nurse
N8	Female	Hospital Oncology nurse, Board member of the Association for Oncology Nursing
N9	Female	Hospital Palliative nurse, also working at the Nursing Management, the Ministry of Health
N10	Female	Hospital clinical nurse specialist
N11	Male	Hospital Palliative nurse
N12	Female	Hospital Palliative and radiotherapy nurse
N13	Female	Senior Nurse, Head of the ambulatory division in a hospital, including Oncology and Hematology clinics
N14	Female	Hospital Palliative nurse
N15	Female	Senior Nurse, Department of professional development in the Nursing Management, the Ministry of Health
N16	Female	Hospital Palliative nurse, breast cancer specialist
N17	Female	Hospital Palliative nurse
N18	Female	Head of the hospital nursing oncology division

**Table 2:** Theme 1—Contribution to the healthcare system.

Quotes
“Knowing how to get a person settled back at home, for example, can reduce hospitalizations. When a patient goes home with instructions and is in contact with a clinical nurse specialist, this will reduce visits to the emergency room and hospitalizations and contributes to the wellbeing of the patient and the family.” (N16) “If there are nurses who know how to respond and do physical examinations and observe the patients’ problems before the physician arrives, that would help. Many times, a physician just reviews the tests and does not have time to physically see the patients and talk to them.” (N4) “We have a clinical nurse specialist in surgery for the emergency room. She does more than half of the work, so the doctor is freed up to do surgery. She makes diagnoses. She sends for imaging. The patient is not delayed and doesn’t have to wait for the physician to come from the operating room.” (N14) “This could certainly provide a solution to the distress and pressure we live with. I don’t think there is competition here. There is enough load on the system.” (P4) “You need a clinical specialist nurse in practically every field. Certainly, in oncology, the patient needs the emotional support aspect. Being with them, the support, is very, very important. Also, a nurse who knows the patient well can give personalized care, tailored to the patient.” (N10) “Nurses complement physicians’ work—and I emphasize: complement not replace. They do not need to replace a physician’s work—they need to complement a physician’s work. The medical profession must be maintained. I come from within the system and understand the constraints of the system. Therefore, I allow myself to say that I do not agree with the [response to the] constraints of the system, that if there is a shortage of physicians, nurses are brought in.” (P6) “The goal of the nursing administration is to promote the nursing profession, not the system. Despite our evidence, as physicians, that she improves patient care.” (P2)

**Table 3:** Theme 2—Contribution to the patient.

Quotes
“Today, oncology is looking towards the community. People live with metastatic disease for many years, and they live in their community. A person can receive chemotherapy or biological therapy with pills in the community, without going to an inpatient department at a hospital or a radiation institute. Also, the population is older, people live longer, and they have underlying diseases as well as cancer. That’s why the treatments are more and more often given in the community. And this will continue.” (N8) “You can reduce referrals to the emergency room. All patients run to the emergency room, but the emergency room is a very difficult experience for the patient. It exposes them to infections, and they wait for many hours. If someone in the community will go to the patient and take care of things that a nurse can do at the patient’s home, it will be great for everyone.” (N3) “No one sees the patient as a whole, all the various aspects related to dealing with his medical condition. And I think that nursing, specifically, is a field that really keeps an overall view of the patient.” (N5) “Many patients in the community fall through the cracks, they are neither here nor there. There are oncology patients in advanced stages, but not yet in hospice or terminal. They need follow-up. A clinical nurse specialist in oncology can provide the solution.” (N3) “The nurse frees me from the secondary things. This does not free me from seeing the patient, from providing treatment and instructions. But it improves service to the patient.” (P5)

**Table 4:** Theme 3—Drawing professional boundaries.

Quotes
“What will this contribute to oncology? They [CNS] will learn how to alleviate pain and symptoms. What can a nurse contribute more than a clinical nurse specialist in palliative care does? Beyond that, someone could only prescribe chemotherapy, and I don’t think anyone [a nurse] would want to take that on herself.” (N9) “Specifically for oncology? I think there is such a shortage of nurses, that this seems to me like a luxury. As it is, there are not enough nurses, in my opinion.” (N16) “It will hurt the quality of treatment. It cuts into the professional authority that until now has been given to physicians. It also hurts the physician’s status and standing. The physicians feel that their authority is being undermined. Instead of making the effort to find, train, and employ enough physicians, they bring in personnel of lower quality to do things that are proper medical functions. The result of all this diminishes the quality of medical services. Maybe it’s in exchange for increased availability, because there are more nurses. But it’s definitely a reduction of quality.” (P6) “The medical profession has only two unique aspects: making a diagnosis and providing treatment. Only a physician can do those things. Whether there is a need for a mid-level practitioner in oncology is a question you should ask oncologists. I think there is. Should that mid-level practitioner be a nurse? In my opinion, no. They should talk to us, the physicians, about this, and it should be done in a way that is cooperative and not adversarial… In many professions, this is not a real need in the system. If there is a real need in the system and we need personnel who are not physicians, we need to create assistant physicians, then we will have an additional profession and will not take the best nursing minds away from nursing and towards medicine, when we already have a shortage in nursing.” (P2) “There can’t be any confusion between professions. Nurses have enough to do. I am not sure they should also be given options that require a broad understanding of the patient. In this case, openness is based on a misrepresentation of the problem of job standards. The problem of standards is that an informed decision is made by the people who control the flow of money. You don’t have to abolish the professional criteria to cover the money that is going to other places.” (P1)

**Table 5:** Theme 4—Additional responsibilities and authority for oncology clinical nurse specialists.

Quotes
“The difference between a nurse in an oncology ward and a clinical nurse specialist is their authority and in-depth learning. [They can do] things that an ordinary nurse doesn’t have the authority to do: prescribe medications, give referrals for tests, make decisions regarding treatment. At the same time, they have excellent psychosocial skills. [They know] how to communicate with families and people in complicated situations, [to deal] with ethical dilemmas, to support a person at the end of life, and to manage a decision-making process in cooperation with the patient and the family.” (N8) “The added value is that she can provide a sense of balance to patients. She will outline the treatment. Today, she receives instructions from a physician. But if she has the whole range of knowledge about the treatments, the indications, she will have room to take independent action. If we have a clinical nurse specialist in oncology, she will need, for example, to have the ability to respond and make a medical decision about starting a new medication.” (P1)

**Table 6:** Theme 5—Conflicts of oncology clinical nurse specialists’ professional status.

Quotes
“I don’t know if the field is ready. How many of the other teams, such as physicians or paramedical teams understand what this role is, what it includes, how to cooperate with that role? Here, I think it might be a little more problematic.” (N2). “I think that even now there is not full implementation of the responsibilities that already exist. There is a lot of complexity around it. I don’t know if I would be involved in expanding the list of responsibilities, but I would be involved in seeing that what is already on the list is carried out.” (N16) “There are physicians who accept it, and there are physicians who have a hard time with it—mostly physicians in the community. Physicians in the hospital love the [clinical nurse] specialists because, for them, this is another significant help in treating the patient.” (N18) “I think physicians also understand that this is important. The future is going in that direction. If the United States already had this thirty years ago, accepting nurse specialists [working] independently is very common there, so there is no reason why it couldn’t happen in Israel. In my case, some raised their eyebrows and said, ‘Who are you, as a nurse, to tell me what to do?’” (N10) “Only if you are weak, then you are afraid of the rise of the nurse. The nurse will not take my place. But she is my right hand.” (P5)

## Data Availability

The data that support the findings of this study are available from the corresponding author.

## Appendix A. Interview Guide

1. Tell me a little about yourself, your current role, and any previous noteworthy roles you performed.
2. What do you know about the role of a clinical nurse specialist in Israel and worldwide? How about developing clinical expertise among nurses, arguments for and against?
3. What do you think is the contribution of a clinical specialist nurse to patients and the healthcare system?
4. In Israel today, there are clinical specialists in nursing in only six fields. Do you think the role of a clinical specialist nurse in oncology is required in Israel? In the hospital? In the community? In both? What do you think its added value is?
5. In your opinion, what should the functions and authority of a hospital oncological nurse include?
6. In your opinion, what should the functions and authority of a community oncological nurse include?
7. In your opinion, does the development of clinical specialists erode the authority and status of doctors?
8. From your experience—how was the role of clinical specialist nurse received by both the nurses and the doctors? Did you feel the resistances and barriers that you had to remove? Can you give examples?
9. Would you like to add anything?
